# Efficacy of Coronary Computed Tomography Angiography Versus Nuclear Perfusion Stress in Preventing Downstream Imaging and Prolonged Inpatient Length of Stay in Low to Medium Risk Patients With Chest Pain

**DOI:** 10.7759/cureus.27326

**Published:** 2022-07-27

**Authors:** Mileydis Alonso, Radhika K Neicheril, Shruti Shettigar, Allen Lavina, Yelenis Seijo de Armas, Avery Carter, Hong Liang, Ashley Alonso, Jared S Piotrkowski

**Affiliations:** 1 Cardiology, Cleveland Clinic Florida, Weston, USA; 2 Internal Medicine, Cleveland Clinic Florida, Weston, USA; 3 Internal Medicine, Florida International University, Miami, USA

**Keywords:** coronary computed tomography angiogram (ccta), coronary artery disease, cad, stable chest pain, nuclear stress test, cta

## Abstract

Background

The first-line imaging for low to medium-risk patients presenting to the emergency department with stable chest pain is often a matter of debate. Chest pain is the second most common presentation to the emergency department. Non-invasive imaging has been useful in assisting in the diagnosis of coronary artery disease.

Aim

The aim of this study is to compare outcomes of Single Photon Emission Computed Tomography (SPECT) Nuclear Perfusion Stress and Coronary Computed Tomography Angiography (CCTA) performed in low to medium-risk patients and how they led to prolonged hospitalization and downstream testing.

Materials and methods

A total of 519 patients were selected for chart review using the following criteria: admitted for chest pain and older than 18 years of age. Those who presented with STEMI (ST-Elevation Myocardial Infarction) or non-(N)STEMI were excluded. Among these patients, four patients were excluded since their initial test was neither a CCTA nor SPECT Nuclear (NM) Perfusion Stress test. Another 30 patients were excluded based on HEART score (a clinical tool to stratify the risk of major adverse cardiac events) >7 and 111 patients with estimated glomerular filtration rate (eGFR) <60 were excluded. A total of 374 patients underwent analysis.

Results

Univariate data analysis of 374 patients demonstrated a higher percentage of patients with HEART scores 0-3 underwent CCTA (51.6% vs. 31.8% p=0.0250) when compared to patients with SPECT NM perfusion. Multivariable logistic regression revealed that the difference in length of stay between SPECT NM perfusion stress and CCTA was significant, patients with the CCTA test were less likely to have a length of stay ≥24 hours (odds ratio {OR}=0.41, p=0.0465) compared to patients with NM perfusion stress test.

Conclusion

This retrospective cohort study demonstrated that patients who underwent CCTA upon chest pain admission were more likely to have a decreased length of stay time to less than 24 hours.

## Introduction

Chest pain evaluations are commonly encountered by physicians daily. According to the 2021 American College of Cardiology/American Heart Association guidelines, chest pain is the second most common presentation to the emergency department accounting for >6.5 million visits [1]. Although the etiologies of chest pain are vast, it is crucial to delineate between serious and benign causes of chest pain. Acute coronary syndrome (ACS) needs to be accurately ruled out when patients arrive at the hospital. Most of the patients with low to moderate risk for acute coronary syndrome still enter a “rule out ACS” classification and get admitted to the hospital to undergo further testing despite a percentage of this population not needing additional testing. This culture promotes prolonged hospital stays and unnecessary testing since low-risk patients with acute or stable chest pain often do not require urgent diagnostic testing for suspected coronary artery disease [1]. Of all emergency department patients with chest pain, only 5.1% will have an acute coronary syndrome and more than half will be found to have a non-cardiac cause [2].

Over the past years, non-invasive imaging has been useful in assisting in the diagnosis of coronary artery disease (CAD) and proving prognostic information when indicated. From 1993 to 2008, the percentage of stress tests that included cardiac imaging increased from 59% to 87%, of which 34.6% did not meet standards of appropriateness, resulting in annual direct costs of nearly $501 million and a projected 491 future cases of cancer due to radiation exposure [3]. Despite the increased utility of noninvasive imaging, no agreement exists on the first appropriate test to evaluate new-onset, stable chest pain. This is partially due to the advantages and disadvantages of “functional” testing with exercise electrocardiography, nuclear stress testing, and stress echocardiography versus “anatomic” imaging with coronary computed tomography angiography (CCTA). This test has been found to have a high accuracy in detecting the severity of CAD, however, the Promise trial showed no difference in all-cause mortality and major complications when comparing anatomical and functional tests [4]. On the contrary, the Scot-Heart trial found computed tomography angiography (CTA) to increase diagnostic certainty, increase the identification of obstructive and non-obstructive coronary artery disease, and eliminate the need for further downstream stress imaging tests [5]. 

Current European and US guidelines recommend considering the pretest probability of CAD when choosing the first-line imaging as this can affect diagnostic accuracy [6-8]. Among patients with acute chest pain and low cardiovascular risk (30-day risk of death or major adverse cardiac events {MACE} <1%), no additional urgent cardiac testing may be needed [9]. Among patients with acute chest pain at intermediate risk (patients without high-risk features and not classified as low risk) and no known CAD, additional testing can include functional testing or anatomic testing [9]. Among patients with known CAD and acute chest pain at intermediate risk, additional testing can include functional testing or CCTA in the setting of non-obstructive CAD; functional testing in the setting of known obstructive CAD; or invasive coronary angiography (ICA) in the setting of known left main disease, proximal vessel CAD, or multi-vessel CAD [9].

Optimizing the use of diagnostic imaging tests in patients with suspected CAD is crucial, given that about two-thirds of invasive coronary angiograms performed in Europe and the United States show no evidence of obstructive CAD, and increasing use of cardiac imaging tests poses a burden on healthcare costs [10-11].

## Materials and methods

We performed a retrospective chart review for chest pain admissions between January 2019 to December 2020. Demographics, medical therapy, medical diagnosis, interventional procedure, complications, and length of stay were analyzed. Patients were filtered through inclusion and exclusion criteria. Selected patients were listed by medical record number (MRN) and Research Electronic Data capture (REDcap, https://www.project-redcap.org/) was used to collect the following data: Age, Gender, Race, BMI (body mass index), HEART score (a clinical tool to stratify the risk of major adverse cardiac events), interventions performed, final diagnosis, and length of stay.

Study population

A total of 519 patients were selected for chart review using the following criteria: admitted for chest pain and older than 18 years of age. Those who presented with STEMI (ST-Elevation Myocardial Infarction) or non-(N)STEMI were excluded. Among these patients, four patients were excluded since the initial test was neither CCTA nor SPECT nuclear (NM) perfusion stress. Another 30 patients were excluded based on HEART score>7 and 111 patients with eGFR <60 were excluded due to no patient in the CCTA group having an estimated glomerular filtration rate (eGFR) <60. A total of 374 patients were analyzed.

Statistical analysis

Univariate analyses including the Chi-square test and Fisher’s exact test were used to assess the differences in characteristics and outcomes between SPECT NM perfusion stress and CCTA, while multivariable logistic regression analysis with a backward elimination method was performed to evaluate the difference in length of stay between SPECT NM perfusion stress and CCTA. In addition, the association between the HEART score and the initial test result was assessed using the Chi-square test and Fisher’s exact test. All data analyses were conducted using SAS version 9.4 (SAS Institute Inc., Cary, NC).

## Results

Univariate analyses (Table 1) showed that six variables (age, HEART score, Hispanic origin, hyperlipidemia (HLD) risk-factor 2, risk-factor 4 {male>45}) were significantly unbalanced between the patients with CCTA test and the patients with SPECT NM perfusion stress. Univariate analysis (Table 1) showed a higher percentage of patients with HEART scores 0-3 underwent CCTA (51.6% vs. 31.8% p=0.0250) compared to patients with SPECT NM perfusion stress. 

**Table 1:** Univariate analysis of the differences in patient characteristics and final outcomes between SPECT Nuclear Perfusion Stress and Coronary Computed Tomography Angiogram SPECT= Single Photon Emission Computed Tomography, p-value= probability value, BMI= body mass index, CHD= coronary heart disease, PCI= percutaneous coronary intervention, ACS= acute coronary syndrome, LOS= length of stay, n= number, %= percentage; HEART score= a clinical tool to stratify risk of adverse cardiac events

Variable	SPECT Nuclear Perfusion Stress (n=343)	Coronary Computed Tomgraphy Angiogram (n=31)	p-value
Age, median (range)	64.0(28.7-91.0)	55.0(18-78)	0.0002
Age (n %)			<0.0001
age ≤ 45	20 (5.8)	8 (25.8)	
age > 45	323 (94.2)	23 (74.2)	
Sex, n (%)			0.4278
Male	219 (63.8)	22 (71.0)	
Female	124 (36.2)	9 (29.0)	
Hispanic, n (%)			0.0148
No	254 (74.1)	29 (93.6)	
Yes	89 (25.9)	2 (6.4)	
Body Mass Index, median (range)	28.7(15.6-81.0)	31.5(21.3-55.1)	0.0623
HEART score, n (%)			0.0250
0-3	109 (31.8)	16 (51.6)	
>4-7	234 (68.2)	15 (48.4)	
CHD Risk Equivalent, n (%)			0.1389
0	185 (53.9)	21 (67.7)	
>0	158 (46.1)	10 (32.3)	
Hypertension, n (%)			0.4684
No	90 (26.2)	10 (32.3)	
Yes	253 (73.8)	21 (67.7)	
Hyperlipidemia, n (%)			0.0132
No	122 (35.6)	18 (58.1)	
Yes	221 (64.4)	13 (41.9)	
Family history, n (%)			0.2320
No	276 (80.5)	28 (90.3)	
Yes	67 (19.5)	13 (41.9)	
Male age >45, n (%)			0.0479
No	147 (42.9)	19 (61.3)	
Yes	196 (57.1)	12 (38.7)	
Female age >55, n (%)			0.8737
No	248 (72.3)	22 (71.0)	
Yes	196 (57.1)	9 (29.0)	
Smoker, n (%)			0.6936
No	234 (70.9)	23 (74.2)	
Yes	100 (29.1)	8 (25.8)	
Result of initial, n (%)			0.9866
Negative	266 (77.6)	24 (77.4)	
Positive	77 (22.4)	7 (7)	
Additional testing, n (%)			0.0633
No	276 (86.5)	23 (74.2)	
Yes	43 (13.5)	8 (25.8)	
PCI, n (%)			0.5563
No	304 (88.9)	29 (93.5)	
Yes	38 (11.1)	2 (6.5)	
Final medical diagnosis, n (%)			<0.0001
Non-cardiac/atypical	232 (67.6)	13 (41.9)	
Non-obstructive CAD	44 (12.8)	14 (45.2)	
ACS/other	67 ( 19.5)	4 ( 12.9)	
LOS in Hours, median (Range)	31 (8-403)	28 (9-219)	0.0489
LOS In Hours, n (%)			0.0322
<24	66 (19.2)	11 (35.5)	
≥24	277 (80.8)	20 (64.5)	

Multivariable logistic regression (Table 2) revealed that the difference in length of stay (LOS) between SPECT NM perfusion stress and CCTA was significant. Patients who underwent CCTA were less likely to have a length of stay over 24 hours (odds ratio {OR}=0.41, p=0.0465) compared to patients with NM perfusion stress test.

**Table 2:** Multivariate Logistic Regression Analysis for the event of Length of Stay ≥24 hours The model included 6 co-variables (age, HEART score, Hispanic, risk-factor 2 (hyperlipidemia {HLD}), risk-factor 4 (male>45), and final medical diagnosis), and finally age (p=0.1868), Hispanic (p=0.9589), risk-factor 2 (p=1976), and risk-factor 4 (p=0.4502 were eliminated by backward elimination method. CCTA= coronary computed tomography angiogram, NM perf stress= nuclear perfusion stress, CI = confidence interval, p-value= probability value, CAD= coronary artery disease, ACS= acute coronary syndrome

Effect	Odds Ratio	95% CI	P-value
Initial diagnosis test (CCTA vs. NM perf stress)	0.41	(0.17-0.99)	0.0465
Heart score (4-7 vs. 0-3)	2.04	(1.19-3.51)	0.0100
Final medical diagnosis			
Non-cardiac/atypical	1		
Non-obstructive Coronary Artery Disease	1.94	(0.83-4.55)	0.1278
Acute Coronary Syndrome/other	3.83	(1.45-10.13)	0.0069

Tables 3-4 show that the HEART score was significantly associated with the result of both CCTA and Nuclear Perfusion Stress Tests. Table 3 depicts the correlation of the HEART score with the combination of negative and positive results of each test, respectively. Table 4 gives a more detailed breakdown of the initial test result and HEART score between the CCTA and Nuclear Perfusion Stress Test separately.

**Table 3:** Overall HEART score and Test Result Correlation for both CCTA and Nuclear Perfusion Test combined p-value = probability value, n= number, %= percentage

	HEART score (0-3)	HEART score (>3)	p-value
Initial test result, n (%)			<0.0001
negative	116 (92.8)	174 (69.9)	
positive	9 (7.2)	75 (30.1)	

**Table 4:** Breakdown of the HEART score and Test Result Correlation with respect to CCTA and Nuclear Perfusion Test n= number, %= percentage, p-value= probability value

Coronary Computed Tomography Angiogram	HEART score (0-3)	HEART score (>3)	p-value
Initial test result, n (%)			0.0373
negative	15 (93.7)	9 (60.0)	
positive	1 (6.3)	6 (40.0)	
SPECT Nuclear Perfusion Stress	Heart score (0-3)	Heart score (>3)	p-value
Initial test result, n (%)			<0.0001
negative	101 (92.7)	165 (70.5)	
positive	8 (7.3)	69 (29.5)	

## Discussion

There is mixed data on the appropriate choice of diagnostic testing in the evaluation of a patient who presents with a chest patient. The HEART Score remains an essential risk stratification score that is useful to clinicians in categorizing patients as low, intermediate, or high risk for a major adverse cardiac event (MACE). The HEART Score has been validated in many trials, both retrospective, and prospective [12-15]. Low-risk patients are classified with a score of 0-3, moderate-risk patients have a score of 4-6, and high-risk patients have a score of 7-10. Based on the validity of the score, low-risk patients are potential candidates for early discharge, and moderate-risk patients are potential candidates for observation and further evaluation. Yet some clinicians are hesitant to discharge low-risk patients without further testing, prolonging observation, and/or hospital admission [16]. This was also shown in our study with prolonged lengths of stay.

The CORE320 multicenter study involved 381 patients with a 59% prevalence of obstructive CAD. The primary endpoint for this study was the accuracy of these tests which was represented by the area under the receiver operating characteristic curve (AUC) for identifying patients with >50% stenosis [17]. The results demonstrated a significantly greater accuracy for CCTA with an AUC of 0.91 vs 0.69 in SPECT. Furthermore, data analysis also found CCTA to have a higher sensitivity of 92% compared to SPECT at 62%, concluding that CCTA had a better sensitivity and diagnostic accuracy than SPECT for detecting angiographic obstructive CAD. This study also showed that CCTA was more predictive of revascularization at 30 days as well [17,18]. Other studies such as the CATCH study found the CCTA to have a positive predictive value of 71% when diagnosing CAD compared to 36% when utilizing stress testing [19]. Trials including the Promise and Scot-Heart trials have compared the effectiveness of CCTA vs stress testing and reported similar near-term effectiveness at 2-3 years follow-up [4,5,20-22]. The Promise trial demonstrated that patients randomized to stress testing had no difference in the primary outcome of death, ACS, or major procedural complications compared to CCTA [4]. The Scot-Heart trial showed similar data findings until additional follow-up analysis was done at the 5-year mark. The addition of CCTA to the standard of care, predominantly consisting of exercising ECG, resulted in a reduction in 5-year CAD death or Acute MI (Myocardial Infarction) when compared to standard care alone [5]. Additional findings of the trial suggested that the use of CCTA resulted in a more correct diagnosis of coronary artery disease than standard care alone, which in turn, led to appropriate therapies, and changes in management resulting in fewer clinical events in the CCTA group compared to the standard group [17]. It is important to recognize that prior studies have claimed that CCTA studies are associated with more invasive testing such as coronary angiography and coronary revascularization [9,10]. But the five years follow-up in the Scot-Heart trial found higher procedures no longer apparent, and on the contrary, found rates of invasive coronary angiography and coronary revascularization higher in the standard-care group than in the CCTA group [23]. 

Another advantage to the CCTA, as shown in our study, was reduced inpatient length of stay and indirect healthcare costs. CCTA examinations were performed in 53 emergency departments, and 50 of them had negative findings on CCTA [19]. The authors discovered that immediate discharge with a negative CCTA reduced length of stay by 80% and charges by one-half compared with functional testing work up - stress myocardial perfusion imaging (MPI) or stress echocardiography [16]. The CT-STAT (Computed Tomography for Systematic Triage of Acute Chest Pain Patients to Treatment) study involved 701 emergency department patients presenting with chest pain, negative biomarkers, and ECGs. These patients were randomized to triaging tests of CCTA vs stress MPI, and it was noted that CCTA reduced the time of diagnosis by 53% and costs of emergency department care by 38% compared with MPI. But the visibility of these advantages is lacking given the underutilization of the CCTA, this is also demonstrated in our study. Overall, the decision of CCTA vs Stress Test should be individually guided, but the preference goes towards CCTA for low-risk patients with a HEART score of 0-3. With this scan, we will be able to focus on improving the diagnostic accuracy of CAD, lowering hospital and patient costs, and further guiding clinical decision-making for the benefit of the patient. 

Study limitations included not considering prior CAD history as a risk factor in the HEART score, and no comparison of the results of subsequent tests that took place after functional testing. Another significant limitation in this study is the wide range of patient age populations that may have augmented a significant imbalance between the number of patients who underwent SPECT NM perfusion stress vs CCTA. This can be attributed to our institution’s culture of an increased propensity to order SPECT NM perfusion stress imaging for chest pain admissions, especially those of older age with more calcified anatomy. Despite this imbalance, no statistical significance was found with regards to increased downstream testing between CCTA and SPECT NM perfusion stress testing. 

## Conclusions

Patients with a HEART score of 0-3 were more likely to undergo SPECT NM perfusion stress testing. This pattern demonstrated that our hospital underutilizes CCTA as an initial test for low-risk patients presenting with chest pain. Furthermore, when looking at the length of stay, we can conclude that patients who underwent CCTA initially were less likely to have a length of stay over 24 hours compared to SPECT NM perfusion stress testing. This can be attributed to the logistical pre-requisites expected for this examination including fasting for at least 3-4 hours prior to the test, avoiding caffeine for 24 hours prior to the test, and the necessary discontinuation of beta-blockers before the test. It is important to note that there was no statistical significance in required downstream testing between SPECT NM perfusion testing and CCTA. In conclusion, CCTA provides accurate data in a timely manner to prevent unwarranted testing and radiation exposure to encourage time-appropriate discharges resulting in reduced indirect healthcare costs. Given the study limitations mentioned above including the differences in age, further randomized control trials are needed to support these findings.
